# Treatment outcomes of injectable thermosensitive hydrogel containing bevacizumab in intervertebral disc degeneration

**DOI:** 10.3389/fbioe.2022.976706

**Published:** 2022-09-19

**Authors:** Qian Chen, Juehan Wang, Qinghong Xia, Lei Wu, Fei Chen, Li Li, Ce Zhu, Miaomiao He, Yulin Jiang, Yong Huang, Hong Ding, Ruibang Wu, Li Zhang, Yueming Song, Liming Liu

**Affiliations:** ^1^ Department of Orthopedic Surgery and Orthopedic Research Institute, West China Hospital, Sichuan University, Chengdu, Sichuan, China; ^2^ Department of Orthopaedics, Affiliated Hospital of North Sichuan Medical College, Nanchong, Sichuan, China; ^3^ Operating Room of Anesthesia Surgery Center, West China Hospital, Sichuan University, West China School of Nursing, Sichuan University, Chengdu, Sichuan, China; ^4^ Histology and Imaging Platform, Core Facilities of West China Hospital, Sichuan University, Chengdu, Sichuan, China; ^5^ The Institute of Clinic Pathology, Sichuan University, Chengdu, China; ^6^ Analytical and Testing Center, Sichuan University, Chengdu, Sichuan, China

**Keywords:** bevacizumab, intervertebral disc degeneration, VEGF, thermosensitive injectable hydrogel, biomaterial

## Abstract

Intervertebral disc (IVD) degeneration (IDD) is a common musculoskeletal disease and its treatment remains a clinical challenge. It is characterised by reduced cell numbers and degeneration of the extracellular matrix (ECM). Nucleus pulposus (NP) cells play a crucial role in this process. The purpose of this study is to explore the role of bevacizumab, a vascular endothelial growth factor (VEGF) inhibitor, in the treatment of IDD through local drug delivery. High expression of VEGF was observed in degenerating human and rat IVDs. We demonstrated that MMP3 expression was decreased and COL II synthesis was promoted, when VEGF expression was inhibited by bevacizumab, thereby improving the degree of disc degeneration. Thus, these findings provide strong evidence that inhibition of VEGF expression by local delivery of bevacizumab is safe and effective in ameliorating disc degeneration in rats. The injectable thermosensitive PLGA-PEG-PLGA hydrogels loaded with bevacizumab is a potential therapeutic option for disc degeneration.

## Introduction

Intervertebral disc degeneration (IDD) is the main cause of low back pain, especially in the elderly population, and brings huge economic and social burden (Francisco et al., 2022). More than 40% of patients with low back pain are caused by IDD. In severe cases it can cause nerve dysfunction in the lower limbs and even incontinence. Therefore, IDD is considered to be the main pathological basis (Barber et al., 2015). Intervertebral disc (IVD) is colloids composed of nucleus pulposus (NP) and annulus fibrous (AF) cells. IDD is characterized by the progressive deterioration of the NP microenvironment, which maintains important biological functions through the production of extracellular matrix (ECM), including type II collagen (COL II), proteoglycan and aggrecan (Xu et al., 2020; Wang et al., 2022). NP requires a hypoxic and avascular microenvironment to maintain normal function. Due to the lack of a complete understanding of the mechanism of IDD, there is still a lack of effective prevention and treatment methods for disc degeneration. (Pan et al., 2018; Wang et al., 2021a).

Currently, drug analgesia is the main conservative treatment for patients with IDD. In severe cases, surgery is necessary. However, these treatment methods are still unsatisfactory due to their side effects and limitations. Hence, researchers have never stopped searching for effective treatment approaches for IDD, particularly the application of regenerative medicine strategies to repair and reconstruct IVD. (Pan et al., 2018; Liao et al., 2021).

Vascular endothelial growth factor (VEGF) plays an important role in tissue homeostasis (Kim et al., 2017). VEGF was found to be highly expressed in degenerating disc tissue, altering the NP microenvironment and accelerating disc degeneration through neovascularization and infiltration. (Lu et al., 2013). In addition, several studies have illustrated that VEGF signaling pathway plays a pivotal role in IDD (Zhang Y et al., 2019; He et al., 2020; Hwang et al., 2020; Wang et al., 2021b; Chen and Jiang, 2021; Ye et al., 2021). VEGF may represent a therapeutic target for IVD degeneration. Bevacizumab is an FDA-approved recombinant humanised monoclonal antibody that is widely used to treat tumours. It works by binding to human vascular endothelial growth factor (VEGF) and blocks its biological activity to inhibit tumour angiogenesis (Garcia et al., 2020; Haunschild and Tewari, 2020; Nakai and Matsumura, 2022). Numerous clinical cases have already proven its efficacy and safety in human patients, which making it easier to translate into clinical applications in other diseases (Garcia et al., 2020).

Nowadays, this anti-tumour drug has been reported to cause serious side effects when systemic administration. To improve the effectiveness of bevacizumab treatment and reduce its side effects. A thermosensitive injectable poly (lactide-co-glycolide)-block-poly (ethyleneglycol)-block-poly (lactide-co-glycolide) (PLGA-PEG-PLGA) hydrogel was utilized for the local controlled-release of bevacizumab to restore degenerative IVDs. The injectable hydrogel has been shown good biocompatibility and degradability and is easily to mix with drugs. It has been used for topical delivery of targeted therapies (Gao et al., 2020; López-Cano et al., 2021). The gel-like characteristic of the injectable hydrogel facilitates injection into the Degenerated area entirely (Pan et al., 2018; Wei et al., 2021). The aim of this study was to explore the Treatment potential of bevacizumab, a VEGF inhibitor, in improving IVD degeneration through local delivery.

## Materials and methods

### Collection of degenerated disc samples

All human degenerated disc samples in this research were obtained from the West China Hospital of Sichuan University, and all trials were approved by the Ethics Committee of Sichuan University. Degenerative IVD samples (n = 10, 5 males and 5 females, aged 48–69 years) were obtained from patients requiring surgical treatment for degenerative spine disease, while non-degenerative controls (n = 5, 2 males and 3 females, aged 42–59 years) were obtained from patients with lumbar fracture requiring surgery. Degenerated IVDs were identified by MRI and histological staining.

Animal experiments were approved by our Animal Protection and Utilisation Committee. Sprague Dawley rats (female, 8 weeks old, 440 g) were obtained from the West China Hospital Animal Centre. The surgical procedure was performed using a previously described method (Han et al., 2008). Briefly, 1% sodium pentobarbital (Sigma Aldrich, St. Louis, MO) was administered intraperitoneally to 48 rats at a dose of 0.3 mg/kg body weight. Pre-puncture radiographs were taken to determine the position of the caudal intervertebral disc (Co7/8) by digital palpation and confirmed by counting the vertebrae from the sacral region in the trial film (Han et al., 2008). After imaging, 48 rats were randomly allocated into four groups: a non-degenerative group (without puncture and without any injection, n = 12), and a degenerative group (with puncture and PBS injection only, n = 12), and a PLGA-PEG-PLGA hydrogel group (with puncture and hydrogel injection, n = 12), a PLGA-PEG-PLGA-Bevacizumab hydrogel group (with puncture and bevacizumab-loaded hydrogel injection, n = 12) for follow-up MRI, histological analysis and immunochemistry at week 8.

### Fixation and histological examination of intervertebral disc tissues

The IVD samples were first fixed with 4% (v/v) paraformaldehyde solution for 1 day and then decalcified with neutral 10% (w/v) ethylenediaminetetraacetic acid solution for 4 weeks. Following this, the samples were dehydrated with gradient alcohol and then embedded in paraffin for tissue entire continuous section. At last, sections of each sample were stained with hematoxylin and eosin (H&E), Safranin O-Fast Green (SO) staining and immunohistochemical analysis.

### Histological score of degenerative intervertebral discs

These sections (7 μm) of IVD were gathered (approximately five slides per sample) for H&E staining and SO staining. Two experienced histological pathologists observed the morphology and number of NP, AF, and endplate (EP) cells through a microscope and evaluated using previously described grading criteria (Kawchuk et al., 2001; Norcross et al., 2003). A normal disc histology score was 5 points; the moderate degenerative disc histology score was 6–11 points, and the severe degenerative disc histology score was 12–14 points.

### Primary culture of rat NP cells

NP tissue was rinsed twice using a PBS containing 1% penicillin/streptomycin and then cut into small pieces. (2–3 mm^3^). The cells were obtained using a type II collagenase (Invitrogen, Carlsbad, CA, USA) digestion method, overnight at 37°C, and the NP cells were suspended in Dulbecco’s modified Eagle’s medium (DMEM). The NP cells cultured in DMEM containing 10% fetal bovine serum (Gibco) and 1% penicillin/streptomycin (Invitrogen) were placed in a 5% CO2 incubator at 37°C. The medium was replaced every 2 days. Cells of the second passage were used for subsequent experiments.

### Bevacizumab and TNF-α treatment *in vitro*

After 24 h of 10 ng/ml TNF-α treatment, rat NP cells were treated with 1 mg/ml bevacizumab for 30 min, and then RNA and protein were extracted for subsequent experiments.

### Immunofluorescence

After being seeded in 24-well plates, cells were treated with different regimens overnight. After incubation, the cells were washed three times with PBS and then fixed with 4% paraformaldehyde for 20 min, then permeabilised with 0.25% Triton X-100 and blocked with 5% bovine serum albumin for 30 min. Next, the cells were incubated with anti-VEGF (1:300; Abcam, ab69479) and anti- COL II antibodies (ab34712, 1:200, Abcam, UK) at 4°C overnight. Subsequently, the cells were washed with PBS followed by incubation with the secondary antibody IgG-rhodamine (1:500 dilution, SAB3700860) (Sigma-Aldrich) and antibody IgG-FITC (1:200 dilution) (Sigma-Aldrich, F0257) for 1 h at ambient temperature, respectively. Nuclei were dyed with 4,6-diamidino-2-phenylindole (DAPI; Beyotime, China). Fluorescence images were observed with a fluorescent microscope (Zeiss Axioplan microscope, Carl Zeiss Microscopy, Thornwood, NY, USA).

### Western blotting

Western blot test was carried out according to standard methods. In brief, the proteins were transferred to the polyvinylidene fluoride (PVDF) membranes (Amersham, Buckinghamshire, UK) immediately after separation through 10% SDS-PAGE gel. Then, the membranes were blocked with 5% nonfat dried milk for 2 h and incubated overnight at 4°C with anti-COL II (ab34712, 1:200; Abcam, UK), anti-VEGF (1:200; Abcam, ab69479), or anti-MMP3 antibody (ab52915, 1:200; Abcam). The membranes were incubated with the secondary antibody at room temperature for 2 h after being rinsed with Tris-buffered saline. The proteins were measured by enhanced chemiluminescence using Bio-Rad Image Lab Software 5.2 (Bio-Rad Laboratories, Hercules, CA, USA).

### RT-qPCR

RNA from rat cells was lysed with TRIzol (Invitrogen, Carlsbad, California, USA) and then reversely transcribed. PCR was implemented with Brilliant SYBR Green QPCR Master Mix (TakaRa) and a Light Cycler instrument (ABI 7900HT). The expression of the compared genes was quantified by verifying the amplification efficiency of the primer pairs. The following primer sequences were used: COL II

Sense5′-GAGTGGAAGAGCGGAGACTACTG-3′,

antisense5′-CTCCATGTTGCAGAAGACTTTCA-3'; MMP3

Sense 5′-TTT​GGC​CGT​CTC​TTC​CAT​CC-3′,

antisense 5′-TTT​GGC​CGT​CTC​TTC​CAT​CC-3'; VEGF

Sense 5′-GCA​CCC​ATG​GCA​GAA​GGA​G-3′,

antisense 5′-ACA​CAG​GAT​GGC​TTG​AAG​ATG​T-3'.

Each experiment was repeated at least three times on different experimental samples.

### PLGA-PEG-PLGA-bevacizumab hydrogel synthesis

Poly (*D,L*-lactic acid-*co*-glycolic acid)-*b*-poly (ethylene glycol)-*b*-poly (*D,L*-lactic acid-*co*-glycolic acid) (PLGA–PEG–PLGA) triblock copolymers were synthesized by ring-opening polymerization of lactide (LA) and glycolide (GA) in the presence of PEG (*M*_n_ 1,500 Da), and stannous octoate was used as catalyst. PLGA−PEG−PLGA triblock copolymers were prepared for subsequent procedure. Concentrations were reported in wt/vol percentages. polymer was weighed about 3 mg and placed in a 4 ml glass bottle, then 10 ml phosphate-buffered saline (PBS) buffer (pH 7.4) was added to make a 30% solution. Then, at 4°C, the mixtures were stirred continuously overnight to ensure that complete dissolution of the polymer. At 4°C, 20% were prepared for stocking by further dilution by adding appropriate volumes of PBS. 0.25 ml of polymer solution was added to the glass vial to prepare the hydrogel. Subsequently, the vial was incubated in a 37°C incubator to trigger the gelation process.

Hydrogels loaded with bevacizumab were prepared by swelling-diffusion method. Bevacizumab (1 mg/ml) was added to PBS containing D-a-Tocopherol polyethylene glycol 1,000 succinate (TPGS), and then, by ultrasonic dispersion and free swelling in continuous contact with 20% (w/v) lyophilized PLGA-PEG-PLGA copolymer at 4°C for 24 h. Finally, at 37°C, the unloaded PLGA−PEG−PLGA hydrogel was eluted by PBS to obtain PLGA−PEG−PLGA- bevacizumab hydrogel.

### Fourier-transform infrared spectroscopy spectra acquisition

Infrared spectra of lyophilized PLGA-PEG-PLGA hydrogels and PLGA-PEG-PLGA-Bevacizumab hydrogels were obtained through using a TGS detector and a ZnO crystal sampling accessory by a Jasco FT-IR-4100 spectrometer (Tokyo, Japan) in cross-task mode. The spectrometer has a detection range of 400-4000cm^−1^ and a resolution of 4cm^−1^. After 100 times scan of each sample, the average spectrum was used for analysis.

### Rheological analysis

Rheological analysis of PLGA-PEG-PLGA hydrogels was carried out at 4°C by using a rheometer (MCR-92; Anton Paar, Graz, Austria). The heating rate was set to 0.5°C/min, the angular frequency (ω) was set to 1 rad/s and the temperature range tested was 10–50°C.

### *In vivo* and *in vitro* evaluation of the biocompatibility of hydrogel

According to the manufacturer’s method, rat NP cells were co-cultured with hydrogel for 1,3,7 days and then tested for live/dead cell activity and CCK-8 cytotoxicity, respectively. (Thermo Fisher; Solarbio, China). After the PLGA-PEG-PLGA-Bevacizumab hydrogel was prepared, the skin was cut in the middle of the dorsum (length 2 cm) and the hydrogel polymer was implanted subcutaneously under aseptic conditions, followed by wound closure. On days 7, 14 and 20 after implantation, the rats were euthanised by inhaling carbon dioxide and the hydrogels was then collected together with the surrounding tissue. These tissue samples were then further used for histological analysis and degradation study.

### *In vitro* release of bevacizumab

PLGA−PEG−PLGA and PLGA−PEG−PLGA-bevacizumab hydrogels were placed in 20 ml of PBS (pH = 7.4) respectively, followed by a water bath at 37°C. Then 3 ml of the supernatant was taken from each solution at different time points for testing and then 3 ml of fresh PBS was added to the original solution. The absorption value of each sample at 260 nm was measured with a UV–Vis spectrophotometer. The concentration of Bevacizumab in the sample solution was calculated via the standard curve and the cumulative release was counted to identify the bevacizumab release profile of this hydrogel delivery system. The measurement wavelength was determined by full-spectrum measurements.

### Treatment effect of the PLGA−PEG−PLGA-bevacizumab hydrogel in a rat model of IDD

To assess the effect of bevacizumab *in vivo*, 8-week-old Sprague Dawley rats from the Sichuan University Animal Center were acquired for animal experiments. In total, rats were randomly divided into four groups: the non-degenerative (NC) group, the degenerative control (DC) group, the PLGA-PEG-PLGA hydrogel group, and the PLGA-PEG-PLGA-Bevacizumab hydrogel group. The rat IDD model was then created in accordance with previous methods. At week 8, the degree of disc degeneration was first assessed by micro-computed tomography (micro-CT) and MRI. All photos were analyzed using ImageJ software. The disc height index (DHI) was measured in accordance with previously described method (Masuda et al., 2005). Next, the rats were euthanised and the IVDs were then extracted for subsequent experiments. Such as: HEstaining, SO staining, immunohistochemistry.

### Statistics analysis

Data were analyzed using SPSS 20.0 (SPSS, Chicago, IL, USA) and presented as the mean ± SD. Analysis of variance or Student’s t-test with the Student–Newman–Keuls (SNK) posthoctest were performed to determine the statistical significance between differences; *p* < 0.05 indicated statistical significance.

## Results

### VEGF is highly expressed in rat and human degenerative intervertebral discs

**FIGURE 1:**
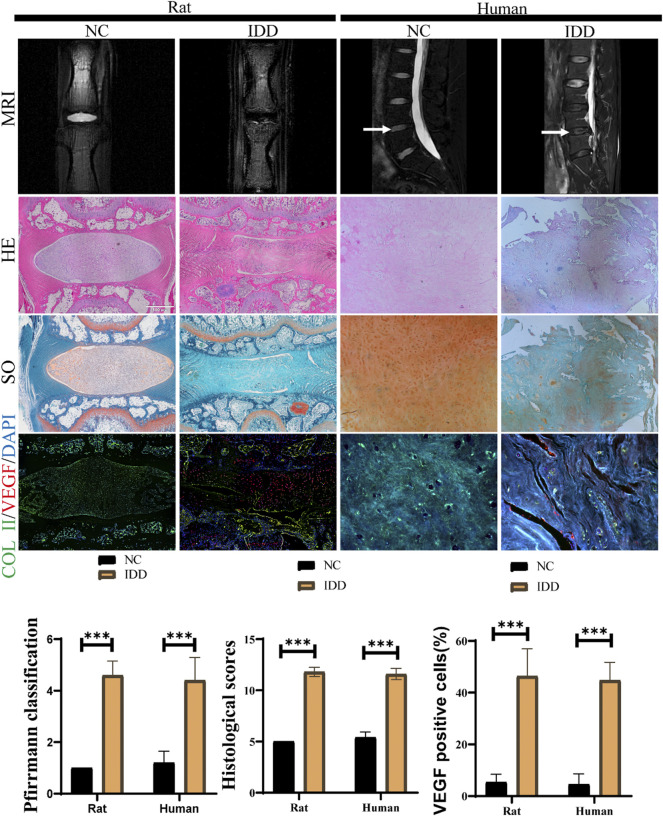
Aberrant VEGF expression in IVDs in rats and humans. **(A)**MRI, H&E staining, SO staining for observation of disc degeneration, and immunofluorescent staining for measurement of VEGF (red) and COL II (green) expression in human and rat IVDs. **(B,C)** The histological scores and MRI grading scores in human and rat IVDs. **(D)** Ratio of VEGF positive NP cell numbers to total NP cells in IVDs of human and rat. data are expressed as mean ± SD (n = 5). *p < 0.05; ***p < 0.01. Scale bar = 500 um.

Safranin O-Fast Green, histological staining and MRI were undertaken to determine the degeneration of rat and human IVDs. In contrast to non-degenerative IVDs, The MRI T2-weighted signal intensity was significantly lower in degenerative IVDs (Figure 1A). By H&E staining, a few chondrocyte-like cells and disordered, hypocellular fibrocartilaginous tissue was observed in IDD of rats. The NP tissue of degenerative IVDs was a loss of structural integrity and lacked extracellular matrix (Figure 1A). In SO staining, the extracellular matrix of normal NPs was cartilaginous and appears orange in colour. However, the degenerating NPs matrix was green in colour because it was fibrous. (Figure 1A). The pfirrmann MRI grading of rat and human degenerative discs was markedly higher than that of the non-degenerative group (Figure 1B). As well as the histological score of rat and human degenerative IVDs above non-degenerative controls (Figure 1C).

The expression levels of VEGF and ECM component COL II in NP cells was observed by immunohistochemistry (Figure 1A). The proportion of positive cells showed that VEGF was notably upregulated in degenerating IVDs in rats and humans and COL II expression was significantly inhibited. (Figure 1D). This indicated that the catabolism was more active in degenerated discs.

### Bevacizumab inhibited the expression of vascular endothelial growth factor and prevented the degeneration of NP cells *in vitro*

**FIGURE 2:**
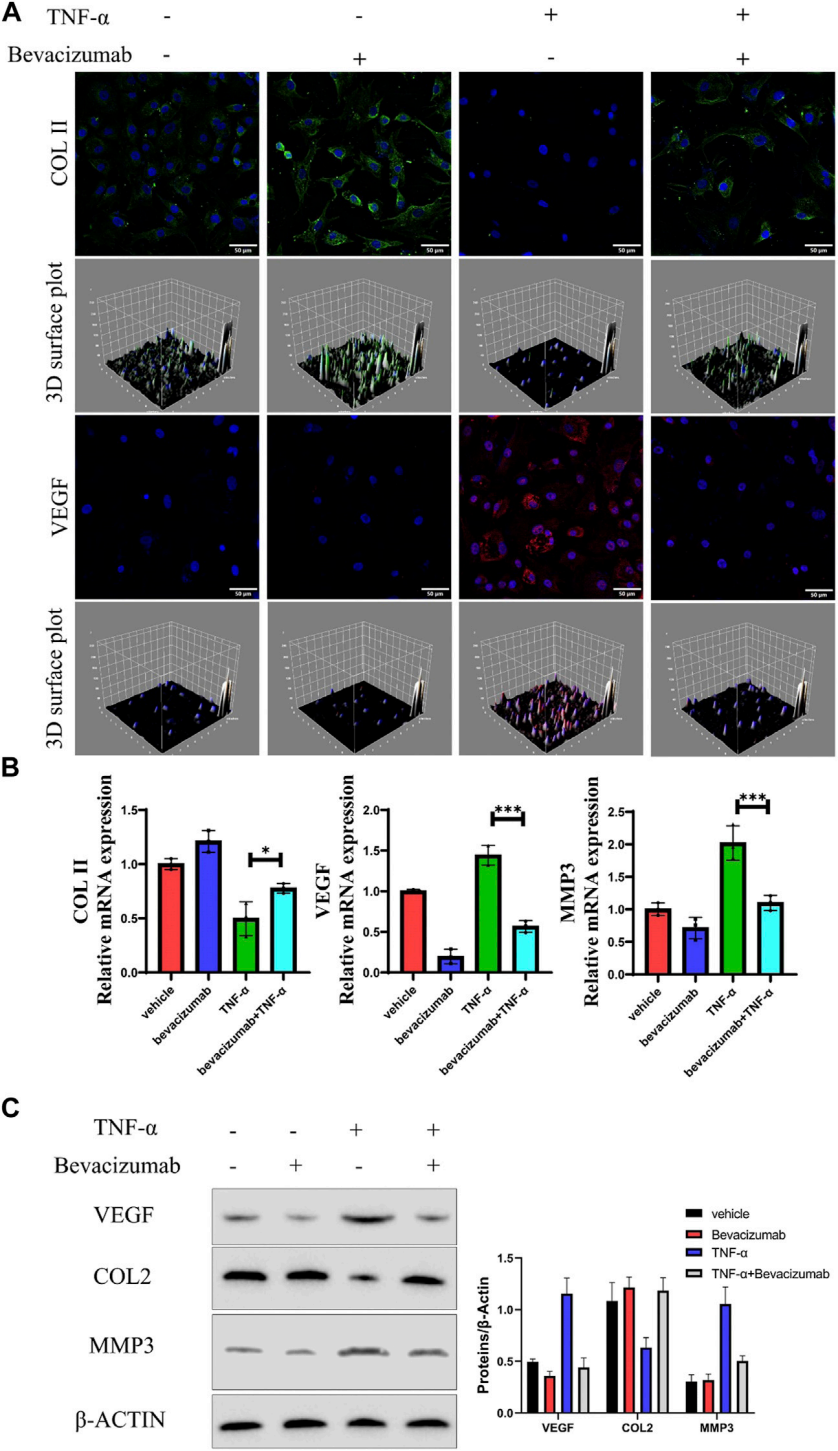
Bevacizumab promotes NP cells anabolism by inhibiting VEGF expression. **(A)** Immunofluorescence analysis of COL II (green) and VEGF (red) production of rat NP cells in 4 groups. Scale = 50 μm. **(B)** The relative expression levels of mRNA in the 4 groups were assessed by RT-qPCR. **(C)** Western blot analysis of VEGF, COL II and MMP13 proteins are displayed in 4 groups. data are presented as mean ± SD (n = 5). *p < 0.05; ***p < 0.01. Scale bar = 50 um.

After confirming that high expression of VEGF may be a potential contributor to IDD, we assessed whether bevacizumab, a specific small molecule inhibitor of VEGF, could inhibit or repair degeneration in NP cells by inhibiting VEGF expression. COL II expression was significantly reduced in degenerating NP cells by Immunofluorescence. Meanwhile, COL expression was partially restored in degenerating NP cells treated with Bevacizumab (Figure 2A). RT-PCR results showed that the results of PCR showed that COL II mRNA expression levels were significantly restored after inhibition of VEGF expression, while the expression levels of MMP3 mRNA were significantly inhibited (Figure 2B). Similar to RT-PCR results, after VEGF expression was inhibited, Western blot results showed that COL II protein expression was significantly increased and MMP3 expression was decreased in TNF-α treated NP cells (Figure 2C). These results suggest that bevacizumab inhibited the degeneration of NP cells by suppressing the expression of VEGF.

### Controlled release of bevacizumab injectable thermosensitive hydrogel with proven biocompatibility

**FIGURE 3:**
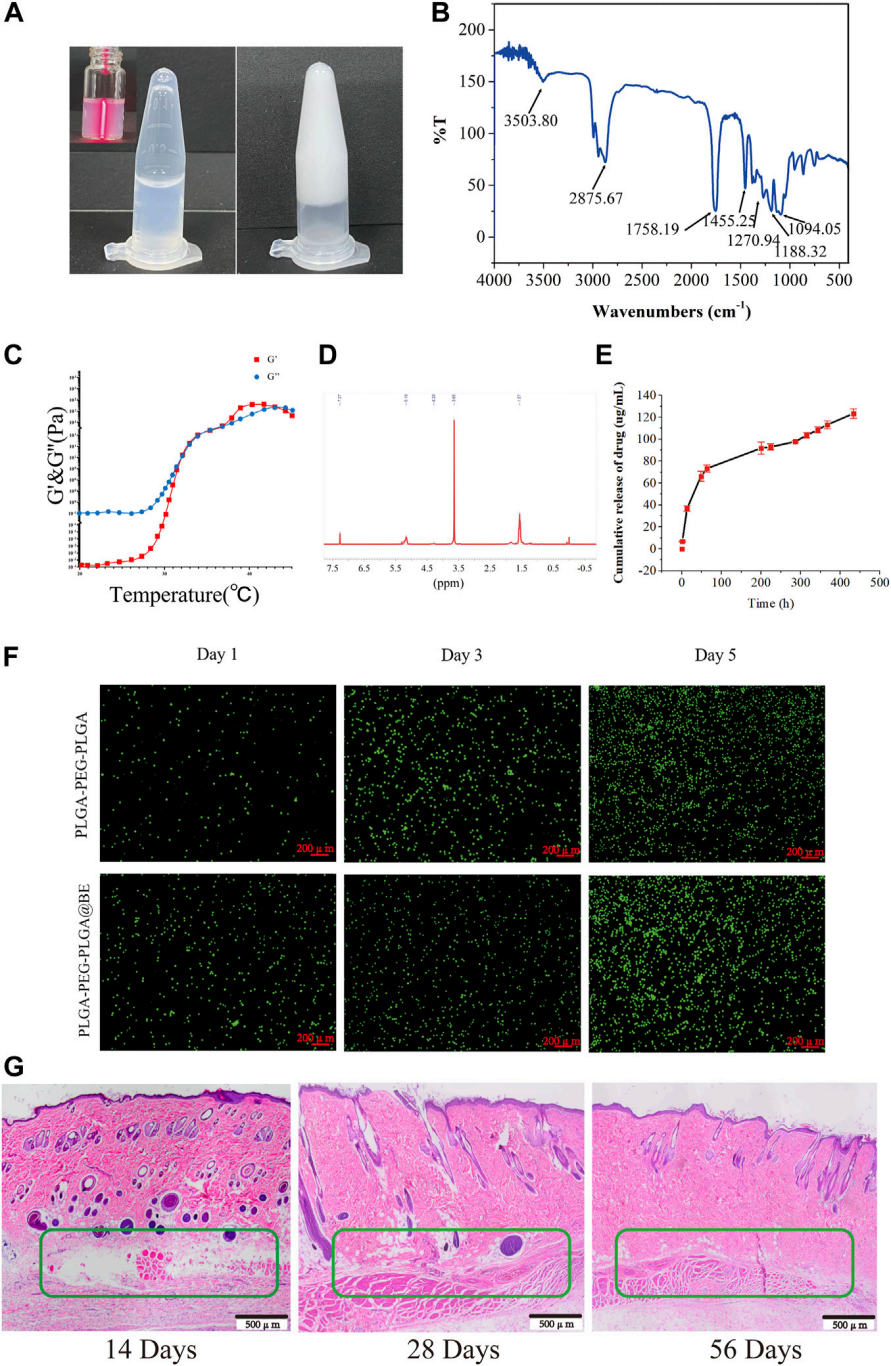
Controlled release of bevacizumab from thermosensitive injectable hydrogels with biocompatibility. **(A)** Sol-gel phase transfer measurement of PLGA-PEG-PLGA copolymers at 37°C using the vial inversion test. **(B)** FTIR transmission spectra of PLGA-PEG-PLGA-bevacizumab hydrogel. **(C)** Storage modulus G′ and loss modulus G″ of the PLGA-PEG-PLGA in PBS (20%). **(D)**The ^1^H NMR spectrum of the PLGA-PEG-PLGA triblock copolymer. **(E)** Release curve of bevacizumab releasing from PLGA-PEG-PLGA hydrogel at 37°C. **(F)** Live/dead dyeing of NP cells in rats incubated on PLGA-PEG-PLGA hydrogels and PLGA-PEG-PLGA-bevacizumab hydrogels on day 1, 3 and 5, respectively. Red fluorescent cells indicate dead cells and green fluorescent cells are surviving. Scale bar = 200 um. **(G)** H&E staining of subcutaneously implanted PLGA-PEG-PLGA-bevacizumab hydrogels on postoperative days 14, 28, and 56. The green box indicates subcutaneous hydrogel. Scale bar = 500 um. data are expressed as mean ± SD (n = 5).

To achieve a safe and effective therapeutic effect, we need to work on the development of a drug delivery system for locally controlled release of bevacizumab. PLGA-PEG-PLGA polymers have been successfully used as drug delivery vehicles due to their excellent biocompatibility and thermosensitive as well as their ability to form hydrogels *in situ*. Therefore, it was chosen for local delivery of bevacizumab (Figure 3A). FTIR analysis confirmed the successful synthesis of PLGA-PEG-PLGA-bevacizumab (Figure 3B). The 20% concentration copolymer is liquid below room temperature and forms a gel as the temperature rises. Similar results for the sol-gel transition temperature were obtained by rheology (Figure 3C). We examined the composition and structure of the copolymers by ^1^H NMR spectroscopy (Figure 3D). The signals of −CH3 and −CH in the LA segment appeared at 1.57 and 5.16 ppm; −CH2 of the GA and EG fractions were observed at 4.29 and 3.65ppm, respectively. At 37°C, release profile of bevacizumab from PLGA-PEG-PLGA hydrogel shows a long drug duration of action (Figure 3E).

To observe the impact of bevacizumab-encapsulated hydrogels on cellular activity at different time points, a live/dead cell assay was performed. After 1, 3 or 7 days of incubation respectively, the cell viability or cell numbers of each group were tested and the results were not significantly different. (Figure 3F). In order to further investigate its biocompatibility, PLGA-PEG-PLGA-Bevacizumab hydrogels were implanted subcutaneously in rats to evaluate degradation rates and local responses with animal tissues, as well as immune reactions with the host. At days 14, 28, and 56, improvements in biocompatibility and integration of the hydrogel *in vivo* were observed (Figure 3G). These results suggest that thermosensitive hydrogel containing bevacizumab is safe and effective for the treatment of IDD.

### Treatment effect of injected bevacizumab-loaded thermosensitive hydrogel in a puncture-induced rat model of IDD

**FIGURE 4:**
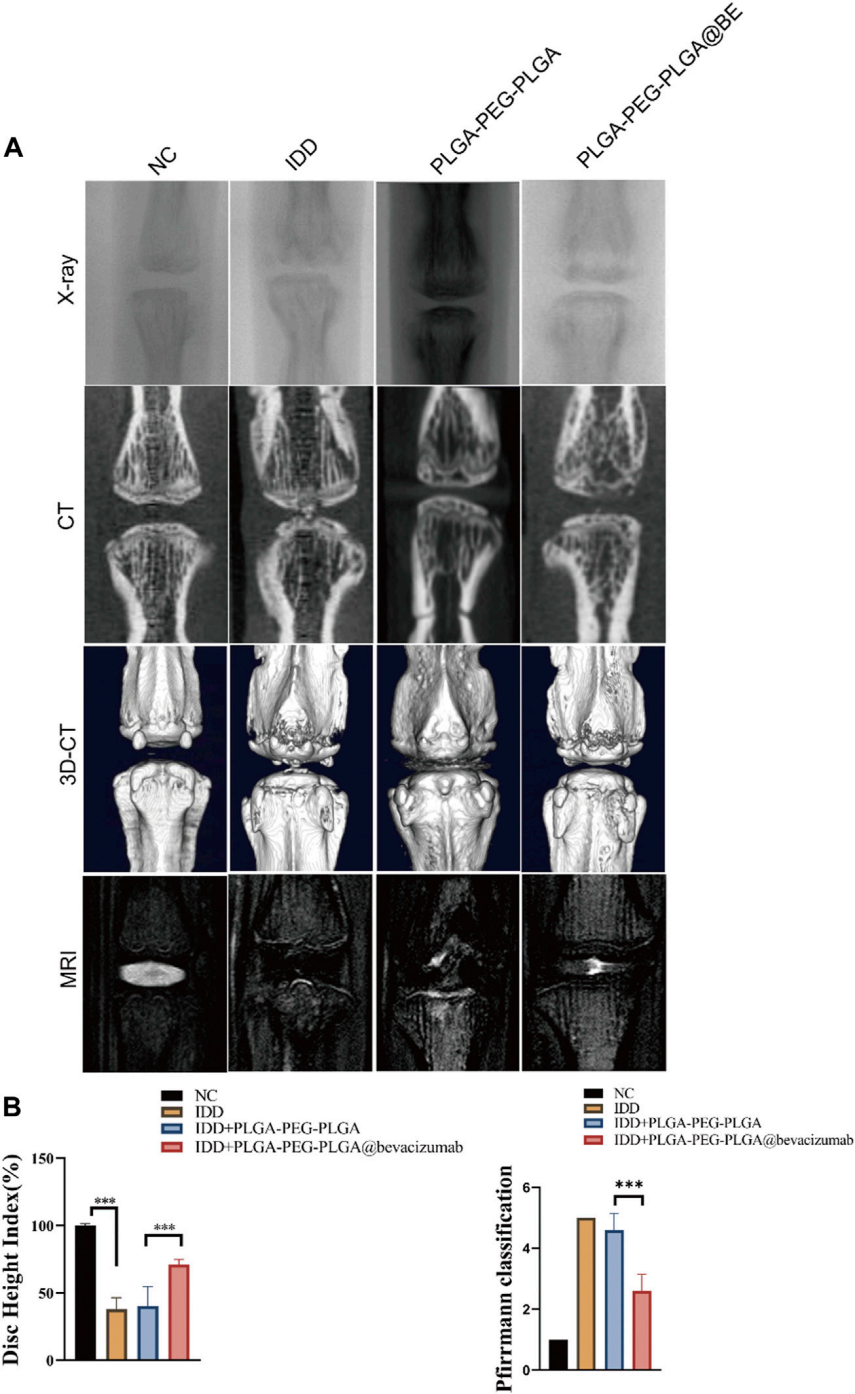
Radiological data of animal experiments by imaging system. **(A)** Representative X-ray, CT and MRI images of the rat’s caudal vertebrae at 8 weeks. **(B)** The change of DHI% in 4 groups at 8 weeks. (C) MRI grading changes in each group at 8 weeks. data are expressed as mean ± SD (n = 5). *p < 0.05; ***p < 0.01.

Puncture-induced rat IDD model was established to assessment the therapeutic effect of PLGA-PEG-PLGA-Bevacizumab hydrogels *in vivo*. At 8 weeks following puncture injury, a significant increase in the number and size of osteophytes was observed in the degenerative control (DC) group. In contrast, less bone formation was detected in the intervertebral space in the non-degenerative (NC) group and the bevacizumab-loaded hydrogel group compared to the DC group (Figure 4A). Changes in the water content of the nucleus pulposus, assessed by MRI T2-weighted signals, can also reliably reflect disc regeneration. At 8 weeks, compared with the DC group, the MRI T2-weighted signal in nucleus pulposus was significantly restored in the bevacizumab-loaded hydrogel group. (Figure 4A). The DHI% (disc height index %) values and MRI grading score were significantly higher in the bevacizumab-loaded hydrogel group than in the DC group (Figure 4B). Thus, the bevacizumab-loaded hydrogel group showed the best protection against IVD, while the hydrogel treatment alone did not have a significant protective effect.

**FIGURE 5:**
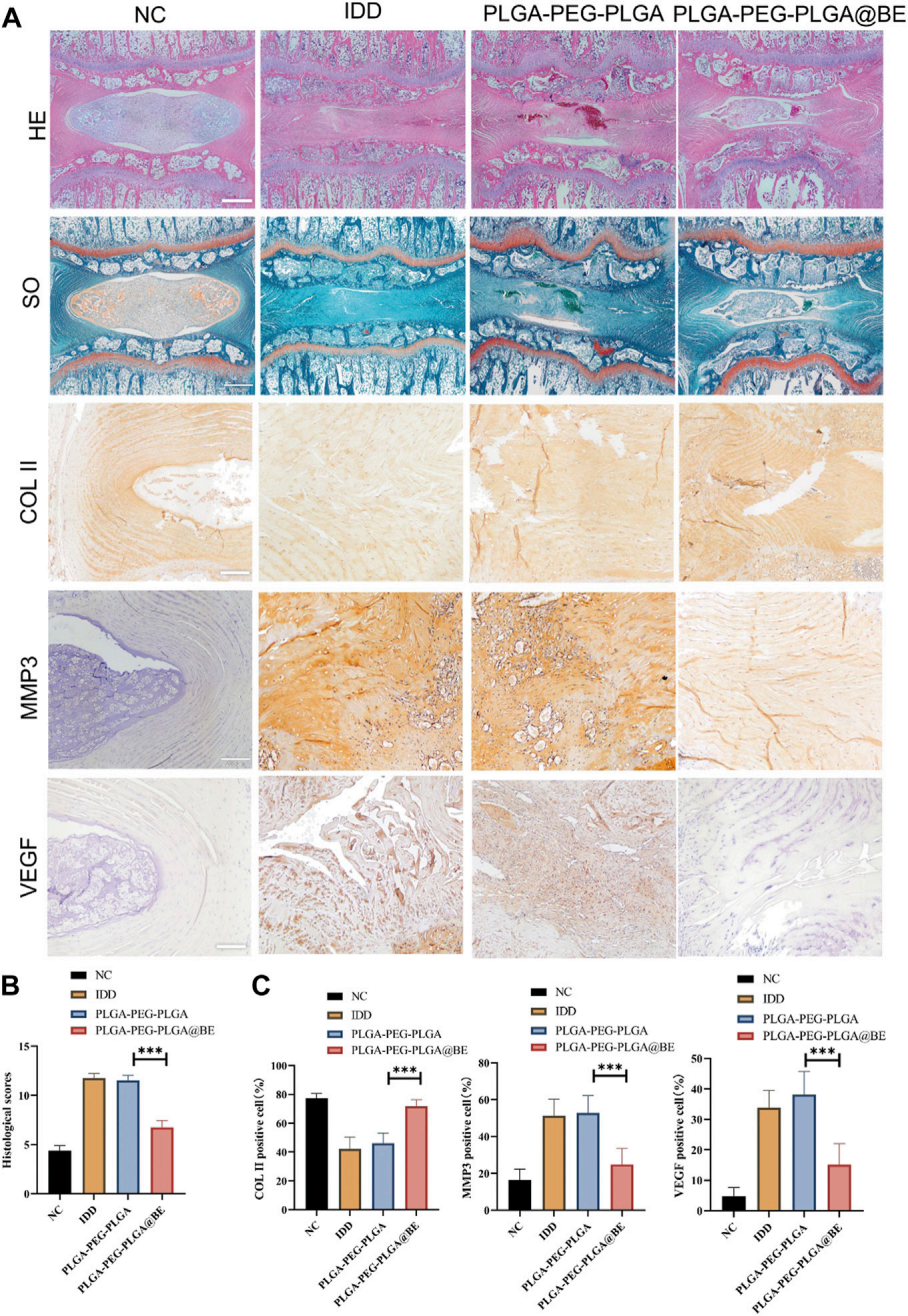
Histological assessment of rat IDD model. **(A)** H&E staining, SO staining, and Immunohistochemistry staining of COL II, MMP3, VEGF. **(B)** Histological grades in week 8 in each group. **(C)**Ratio of COL II, MMP3, VEGF positive NP cell numbers to total NP cells in 4 groups. data are presented as mean ± SD (n = 5). *p < 0.05; ***p < 0.01. Scale bar = 200 um.

H&E staining confirmed that bevacizumab-loaded hydrogel played a protective role in NP degeneration and deterioration of IVD structure. In DC group and hydrogel-only group, NP structure was obviously lost, and there was no visible demarcation between AF and NP. However, in the bevacizumab-loaded hydrogel group, the NP cells were slightly degenerated, and although the intact structure of the NP tissue was somewhat disrupted (Figure 5A). With SO staining, the normal extracellular matrix was orange, but the severely degenerated fibrous tissue stained green. In the bevacizumab-loaded hydrogel group, plenty of orange extracellular matrix was found in the NP tissue with only Mild degeneration (Figure 5A). Immunochemical staining revealed an upregulation of COL II and a downregulation of VEGF, MMP3 in the bevacizumab-loaded hydrogel group, compared to what was observed in the DC group and hydrogel-only group (Figure 5A). The histological score in the bevacizumab-loaded hydrogel group was obviously lower than in DC group and hydrogel-only group, with a relatively mild degree of IDD (Figure 5B). The ratio of positive cells revealed that COL II was upregulated in the bevacizumab-loaded hydrogel group, whereas VEGF, MMP3 was downregulated, compared to DC group and hydrogel-only group (Figure 5C). Combined with the results of our *in vitro* study, these findings suggest that PLGA-PEG-PLGA hydrogels loaded with bevacizumab improve IDD by modulating the expression of VEGF and MMP3 in a rat model of IVD degeneration through local delivery of the drug.

## Discussion

IDD is one of the leading causes of impaired movement and low back pain. Because the underlying molecular mechanism of IDD is still unclear, the clinical treatment mainly aims to relieve the symptom instead of targeting IDD directly (Francisco et al., 2022; Wang et al., 2022). In this study, a new strategy was established to treat IDD by the controlled release of bevacizumab from thermosensitive hydrogels, which was shown not only to inhibit the degradation of cartilage matrix, but also to promote the synthesis of COL II. These findings suggest the local administration of bevacizumab for the treatment of disc degeneration has potential clinical application.

Some studies have observed a relationship between VEGF and chondrocytes in terms of physiology and pathology (Chen et al., 2019; Qian et al., 2021; Xiao et al., 2021; Xiaoshi et al., 2021), but there was a lack of relevant studies in IVD. We found that VEGF is highly expressed in IDD of humans and rats. VEGF is an important factor in promoting angiogenesis in both physiological and pathological conditions. High expression of VEGF was associated with vascular infiltration in tissues. Some studies have found that High expression of VEGF is a major factor contributing to angiogenesis in NP tissue (David et al., 2010; Lee et al., 2011; Lu et al., 2013; Binch et al., 2015). Angiogenesis during disc degeneration is thought to be the main cause of low back pain (Pohl et al., 2016; Melincovici et al., 2018; Azarpira et al., 2021). In addition, our study also found that MMP3 expression was significantly reduced following inhibition of VEGF with bevacizumab. Sahin et al. (Sahin et al., 2012) found that VEGF promotes angiogenesis through the induction of matrix metalloproteinase-3 (MMP3) expression, which in turn attenuates tendon biomechanical properties in degenerative tendon disease. It has also been shown that MMP3 promotes the catabolism of extracellular matrix during IDD (Zhang B et al., 2019; Zhao et al., 2020; Song et al., 2021).

Bevacizumab specifically binds VEGF and impedes blood vessel growth and formation. It has been widely used in oncology patients (Garcia et al., 2020; Haunschild and Tewari, 2020; Nakai and Matsumura, 2022). There have been a number of studies applying it to age-related macular degenerative diseases (Gil-Martínez et al., 2020; Weinstein et al., 2020). Our results revealed that inhibition of VEGF with bevacizumab protects IVDs from degeneration. To reduce the side effects associated with the systemic administration of Bevacizumab, such as gastrointestinal perforation, wound healing complications, congestive heart failure, bleeding, the injectable thermosensitive PLGA-PEG-PLGA hydrogel is used as a vehicle for local release of the drug in the treatment of IDD. As a well-established thermosensitive hydrogel, PLGA-PEG-PLGA hydrogel has been used in previous study for the treatment of IDD (Zou et al., 2013). In addition, unique structure of the hydrogel has been shown to be a good carrier for small molecule drugs (Bakhaidar et al., 2019; Chan et al., 2019). When mixed with bevacizumab, the hydrogel can be injected into the NP tissue through a small diameter needle, thus reducing the chance of annulus fibrous damage.

Due to its temperature sensitivity, it forms gel within a few seconds after injection into IVD, thus reducing the risk of drug leakage. Moreover, the characteristics and excellent biocompatibility of the PLGA-PEG-PLGA hydrogel render it an effective and safe drug delivery system (Yu et al., 2013; Dimchevska et al., 2017). The results of our animal experiments showed that the PLGA-PEG-PLGA hydrogels containing bevacizumab had a remarkable effect on the maintenance of the disc height, synthesis of the COL II, and integrity of the disc structure. Nevertheless, without the help of the drug carrier, small molecule drugs are prone to leakage. It was difficult to maintain adequate drug concentrations in lesion areas of the IVD and may cause adverse events. When PLGA-PEG-PLGA hydrogel was used alone, it did not produce a therapeutic effect in the degenerated discs due to insufficient biological effect. Our results, which are identical to those of previous studies, confirm this again (Zou et al., 2013; Frauchiger et al., 2017). The therapeutic effect of bevacizumab alone or hydrogel alone was not ideal, which also confirmed the necessity and effectiveness of demonstrating the necessity of using the PLGA-PEG-PLGA hydrogels drug delivery system in IDD treatment. The degenerative disc did not recover completely after treatment with bevacizumab-encapsulated hydrogels, which may be due to the complex microenvironment of the degenerative disc. Inhibition of VEGF alone is not sufficient to address all issues. The molecular basis and targeting factors of IDD need to be further explored.

There are some limitations to our work, for example, the rat IDD model established by caudal disc puncture, which differs from human IDD. Not only that, the main limitation is the biological differences between rat and human IVD. Compared with human NP cells, rat NP cells were more spinal cord cells than chondrocytes; thus, rat NP cells may have differential response to treatments (Bang et al., 2018; Dudli et al., 2018). Moreover, because rats are reptiles, unlike humans, their intervertebral discs do not bear the longitudinal load of their body weight. Therefore, more studies are needed on bipeds that are biologically and biomechanically similar to humans IVDs, so that the results can be applied to humans.

## Conclusion

In conclusion, our findings provide evidence that bevacizumab, an FDA-approved drug, plays a protective role in disc degeneration by inhibiting VEGF expression and promoting extracellular matrix synthesis. These results believe that bevacizumab, administered topically through the injectable thermosensitive PLGA-PEG-PLGA hydrogel drug delivery system, has a potential therapeutic effect on IDD.

## Data Availability

The original contributions presented in the study are included in the article/Supplementary Material, further inquiries can be directed to the corresponding author.
